# *Campylobacter jejuni*-Related Myocarditis: A Case Report and Review of the Literature

**DOI:** 10.3390/jcm13247551

**Published:** 2024-12-11

**Authors:** Virginia Zouganeli, Christos Kourek, Vasiliki Bistola, Maria Mademli, Ioannis Grigoropoulos, Konstantinos Thomas, Sotirios Tsiodras, Gerasimos Filippatos, Dimitrios Farmakis

**Affiliations:** 1 Department of Cardiology, Athens University Hospital Attikon, National and Kapodistrian University of Athens Medical School, 124 62 Athens, Greece; virginia_noa@yahoo.gr (V.Z.); vasobistola@yahoo.com (V.B.); gfilippatos@gmail.com (G.F.); 2 Department of Cardiology, 417 Army Share Fund Hospital of Athens (NIMTS), 115 21 Athens, Greece; chris.kourek.92@gmail.com; 3 Second Department of Radiology, Athens University Hospital Attikon, National and Kapodistrian University of Athens Medical School, 124 62 Athens, Greece; mariamademli@yahoo.gr; 4 Fourth Department of Internal Medicine, Athens University Hospital Attikon, National and Kapodistrian University of Athens Medical School, 124 62 Athens, Greece; grigoropoulosioannis@gmail.com (I.G.); costas_thomas@yahoo.com (K.T.); tsiodras@med.uoa.gr (S.T.)

**Keywords:** *Campylobacter jejuni*, gastroenteritis, infection, myocarditis, troponin

## Abstract

*Campylobacter jejuni*, a common cause of gastroenteritis worldwide, has also been associated with rare extraintestinal infections, including myocarditis. We report a unique case of a 24-year-old male who presented with febrile diarrhea and acute chest pain. Diagnostic investigations revealed elevated cardiac troponin levels, normal electrocardiography findings, and myocardial inflammation on cardiac magnetic resonance imaging, confirming the diagnosis of acute myocarditis. Stool cultures identified *Campylobacter jejuni* as the causative agent. The patient was managed with supportive care, including hydration and electrolyte replacement, and a three-day course of azithromycin (500 mg daily). He achieved a full recovery and was discharged after eight days, with subsequent follow-up demonstrating the complete resolution of myocardial dysfunction. This case emphasizes the need to consider *Campylobacter jejuni*-related myocarditis in the differential diagnosis of young patients presenting with chest pain and diarrhea, along with an overview of its diagnostic and therapeutic challenges.

## 1. Introduction

Myocarditis can develop as a complication of various infectious diseases with a broad range of pathogens including viruses, bacteria, fungi, protozoa, and helminths. The most frequently implicated viruses in Europe and North America are parvovirus B-19 and human herpesvirus 6 (HHV 6), followed by Epstein–Barr virus, enterovirus, human cytomegalovirus, adenovirus, coxsackie and, most recently, severe acute respiratory syndrome coronavirus 2 (SARS-CoV-2) [1]. The bacterial causes of myocarditis are less common in western countries, with *Staphylococcus aureus* and *Streptococcus* spp. being the most frequent, followed by *Salmonella, Shigella*, and *Clostridium* spp. [2]. The presentation of acute myocarditis ranges from a lack of symptoms and mild self-limiting illness to cardiogenic shock requiring mechanical circulatory support and cardiac transplantation and/or sudden cardiac death in adolescents and young adults, depending on the cause and the extent of myocardial damage.

*Campylobacter jejuni* is a helical-shaped, non-spore-forming, Gram-negative, microaerophilic, nonfermenting motile bacterium that is capable of changing into a coccal form when exposed to atmospheric oxygen [3]. It is considered one of the most common causes of gastroenteritis worldwide, but extraintestinal infection can also occur, including some rare cases of cardiac involvement, the pathophysiological mechanisms of which remain unclear.

We hereby report a case of *Campylobacter jejuni*-related myocarditis in a 24-year-old man presenting with febrile diarrhea and acute chest pain and compare our therapeutic approach with the few previous worldwide similar case reports available.

## 2. Case Report

A 24-year-old man with no significant medical history presented to the emergency department with acute-onset chest pain that woke him up, describing it as a non-radiating pressure-like pain lasting for a few minutes. He also reported multiple episodes of watery diarrhea that developed two days prior to hospital admission and was associated with a fever of 39.7 °C. He denied eating undercooked meat or unpasteurized milk; he also denied recent ill contacts. He had a history of anxiety disorder, receiving fluvoxamine, with no family history of premature cardiac disease.

**Figure 1:**
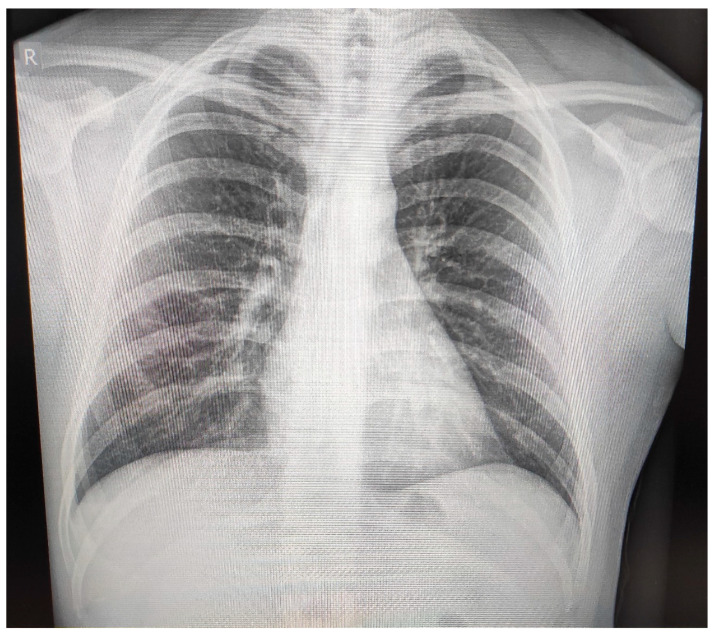
Chest radiograph of the patient. R, right.

**Figure 2:**
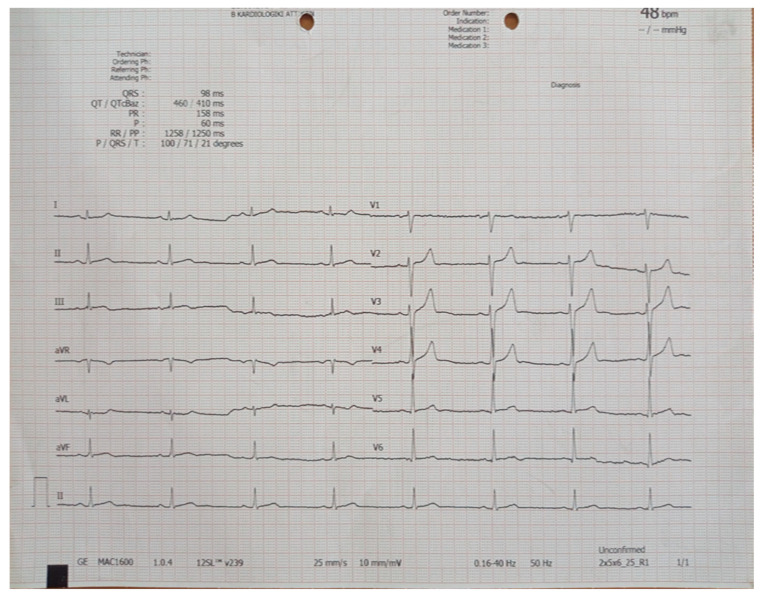
Electrocardiogram of the patient during his admission to the emergency department.

On his arrival at the emergency department, he was alert, with a temperature of 37.7 °C, blood pressure of 130/75 mmHg, regular pulse rate of 68 beats per minute, and oxygen saturation of 99% on room air without dyspnea. Physical examination revealed an alert man. The lung fields were clear on auscultation and a cardiovascular exam revealed a regular rate and rhythm with a normal S1 and S2 and no added sounds. His abdomen was soft, with mild tenderness. Chest radiography revealed no abnormal findings (Figure 1). Electrocardiography (ECG) showed a normal sinus rhythm with nonspecific repolarization disturbances (Figure 2).

Full blood count revealed a hemoglobin level of 15.7 g/dL, a white blood cell count of 8.82 K/μL, and a platelet count of 164 K/μL. Biochemistry investigations revealed a sodium level of 137 mmol/L, a potassium level of 4.7 mmol/L, and a creatinine level of 1.11 mg/dL. The C-reactive protein level was 92.9 mg/L (normal range, 0–6 mg/L). The creatine kinase (CK) and CK-myocardial band levels were 337 U/L and 11.4 ng/mL (normal range, <4.94 ng/mL), respectively. His initial B-type natriuretic peptide level was normal, at 16.8 pg/mL. The high-sensitivity cardiac troponin T (hs-cTnT) was significantly raised to 191 pg/mL (normal reference range, <14 pg/mL). A nasopharyngeal SARS-CoV-2 nucleic acid test and upper respiratory tract syndromic multiplex polymerase chain reaction (PCR) were negative. Blood cultures were negative. A stool culture was performed to isolate *Campylobacter jejuni* using selective media and incubation conditions optimized for the organism’s microaerophilic requirements, following Clinical and Laboratory Standards Institute (CLSI) guidelines [4]. Stool samples were inoculated onto *Campylobacter*-selective agar containing antibiotics to suppress competing flora. Plates were incubated at 42 °C in a microaerophilic atmosphere (5% O_2_, 10% CO_2_, and 85% N_2_) for 48 h. Characteristic colonies were identified based on morphology, Gram staining, and oxidase positivity, with final confirmation using biochemical testing.

**Figure 3:**
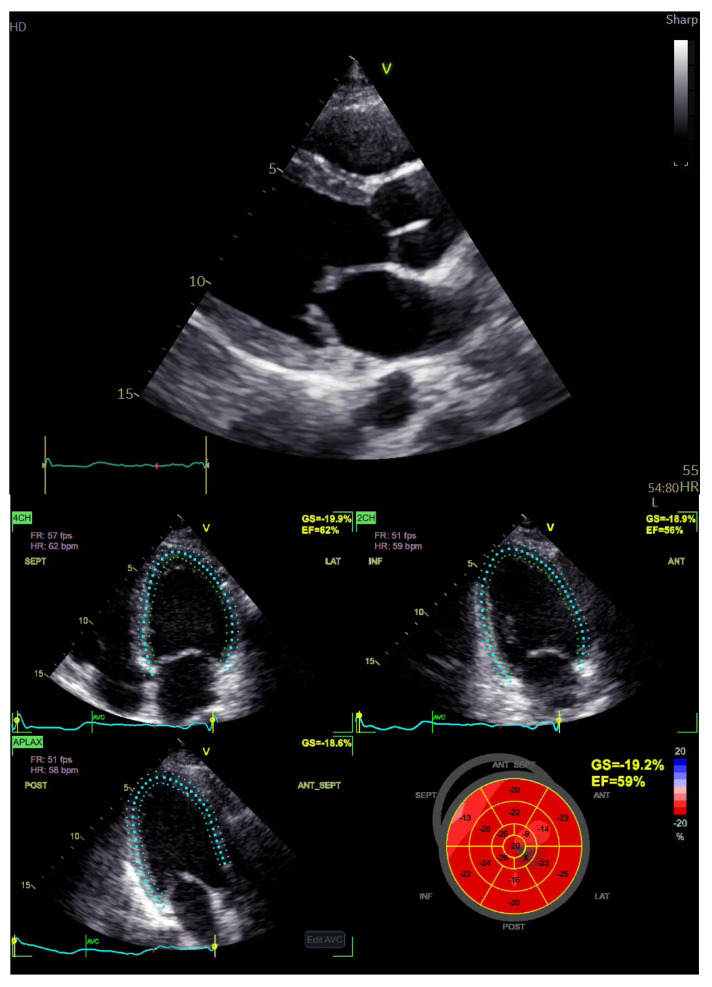
Transthoracic echocardiography findings in the patient, showing normal left-ventricular dimensions and contractility; upper panel, parasternal short-axis view; lower panel, left-ventricular ejection fraction and global longitudinal strain estimations. GS, global strain; EF, ejection fraction; 4CH, 4−chamber view; APLAX, apical long axis; 2CH, 2−chamber view; HR, heart rate; FR, frequency; SEPT, septal; LAT, lateral; INF, inferior; ANT, anterior; POST, posterior; ANT_SEPT, anteroseptal.

Transthoracic echocardiography revealed a left-ventricular ejection fraction (LVEF) >55% with no regional wall motion abnormalities and no pericardial effusion (Figure 3).

**Figure 4:**
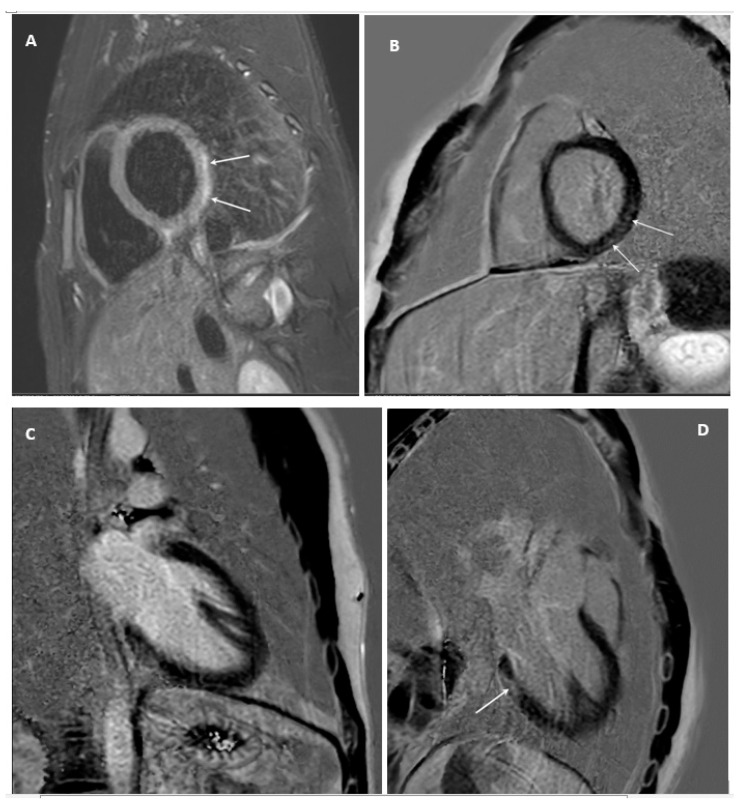
Cardiac magnetic resonance images of the patient: (**A**) short-tau inversion-recovery (STIR) images of the short axis (SA) view demonstrating mid-myocardial increased signal intensity at the basal posterolateral and basal posterior segments, indicative of edema. Late gadolinium enhancement (LGE) images of the (**B**) short axis (SA), (**C**) horizontal long axis (LA), and (**D**) 4-chamber view (4CH), showing increased signal intensity at the same segments with mid-myocardial distribution. The findings are consistent with acute myocardial inflammation.

Cardiac magnetic resonance imaging revealed a midmyocardial increased signal intensity in STIR (short-tau inversion recovery) sequences at the basal posterior and posterolateral segments, indicative of edema, while late gadolinium enhancement (LGE) images of the short axis (SA), horizontal long axis (LA), and 4-chamber view (4CH) demonstrated increased signal intensity at the same segments with midmyocardial distribution. The findings were consistent with acute myocardial inflammation (Figure 4).

During his hospitalization, patient remained asymptomatic, hemodynamically stable, and fever-free. Within the first several days, he presented episodes of diarrhea that lasted up until the fourth day. Continuous telemetry monitoring showed no arrhythmias. The troponin level was elevated to 314 pg/mL on the third day and declined thereafter. The treatment was supportive with hydration and electrolyte replacement. Moreover, he completed a three-day course of 500 mg of azithromycin q.d. while in the hospital. Azithromycin was prescribed empirically, as antimicrobial sensitivity testing of the *Campylobacter jejuni* isolate was not initially performed. Empirical therapy was guided by current clinical guidelines and the high efficacy of azithromycin against *Campylobacter jejuni* infections, as supported by the literature. Specifically, CLSI guidelines recommend sensitivity testing for pathogens when treatment failure is suspected or in cases involving multidrug resistance; however, this was deemed unnecessary in the present case due to the patient’s rapid clinical improvement and the pathogen’s typical susceptibility profile to macrolides [5].

After 8 days of hospitalization, the patient was discharged from hospital with a full recovery. Cardiac enzymes were decreased to normal, and an ECG did not present abnormal findings. Reassessment with blood sample tests after 5 days, ambulatory ECG monitoring and new echocardiography after a month, and cardiac MRI after 6 months were recommended for his follow-up. Moreover, the patient was advised to avoid high-intensity physical activity for at least 6 months after discharge. Follow-up 1 month after admission, including ECG and echocardiography, depicted normal myocardial function.

## 3. Discussion

The *Campylobacter* genus is one of the most common causes of infectious enterocolitis worldwide, with an annual incidence of approximately 1% [6]. *Campylobacter jejuni* infections are common but rarely lead to severe symptoms outside of the gastrointestinal tract. Besides direct bacterial invasion, molecular mimicry-related auto-immune myocarditis could be also caused by *Campylobacter* species [7]. *Campylobacter jejuni*-related myocarditis is a very rare clinical incidence in patients with acute myocarditis.

Patients with myocarditis may present a variety of symptoms, from mild dyspnea or chest pain to severe ventricular arrhythmias and cardiogenic shock and death [8]. *Campylobacter jejuni*-associated myocarditis may be suspected when chest pain with elevated cardiac enzymes occurs shortly after an episode of diarrhea and fever [9]. Endomyocardial biopsy (EMB) has been proposed as the gold-standard diagnostic method for myocarditis. However, due to its invasive nature, the high levels of clinical experience required, and its lack of sensitivity, most clinicians avoid it. It is usually recommended in fulminant and giant cell myocarditis or acute heart failure resistant to conventional treatment [10]. Other nonspecific diagnostic methods, including elevated cardiac enzymes and inflammatory indices such as hs-CRP, troponins Tand I, CK-MB, CPK, leukocyte counts, and ECG abnormalities, as well as more specific noninvasive imaging techniques, including cardiac MRI with gadolinium enhancement, provide a diagnosis with higher accuracy [6].

**Figure 5:**
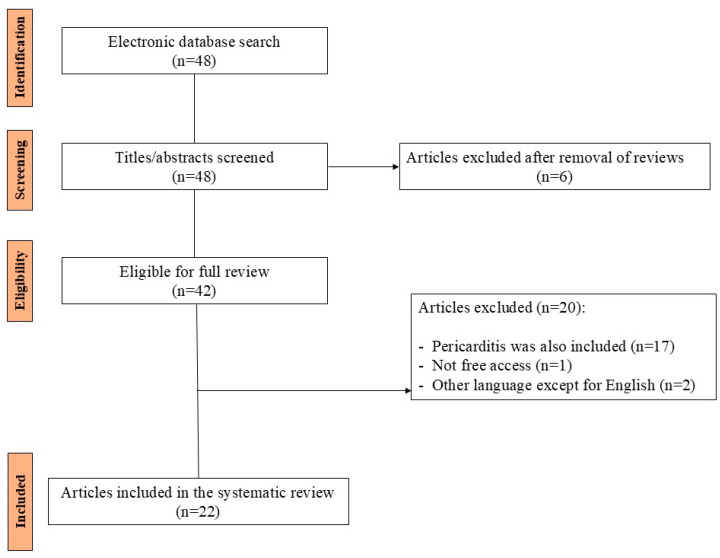
PRISMA flowchart showing the selection of the articles included in the systematic review.

We performed a search on PubMed using the terms “*Campylobacter jejuni*” and “myocarditis”. Our search revealed 46 articles published from 1980 to 2024 (Figure 5). From these articles, we collected case reports that only included patients with myocarditis associated with *Campylobacter jejuni*. We excluded articles including patients with perimyocarditis and endocarditis, patients with other types of *Campylobacters*, articles that were in languages other than English, and articles that were not free. We found a total of 25 cases of *Campylobacter jejuni*-related myocarditis that have been described in the literature, from 22 articles [9,11,12,13,14,15,16,17,18,19,20,21,22,23,24,25,26,27,28,29,30,31].

We highlight, in detail, the baseline characteristics, troponin levels, imaging findings, antibiotics, and outcomes of all case reports of *Campylobacter jejuni*-related myocarditis found in the literature in Table 1. Ages ranged from 13 to 55 years, with the mean age being 28 years. In all case reports, patients were male (96%) except for one female patient. Troponin levels and/or cardiac enzymes including CPK or cardiac MB isoenzyme of CPK were usually elevated during hospital admission, while ECG showed ST elevation or/and conduction abnormalities in most cases [11,12,15,17,19,21,22,23,24,25,26,27,28,29,30,31]. Echocardiography findings demonstrated normal or decreased systolic left-ventricular function with hypokinesis in specific walls in the majority of cases, with a single study showing significant left-ventricular impairment (LVEF 30%) [27]. Cardiac MRI was performed in half of the case reports, with the main findings including late gadolinium enhancement in specific walls, myocardial edema, or small pericardial effusion. As far as the therapeutic approach was concerned, the most frequently used antimicrobial agents were azithromycin, ciprofloxacin, erythromycin, and clarithromycin, while agents not recommended against *Campylobacter*, such as metronidazole, meropenem and gentamycin, were additionally used in some cases. In eight cases, no antimicrobial agents were used [11,12,13,14,17,18,25,29,31]. Regarding clinical outcomes, patients were discharged from hospital within the first week of their admission, and the majority fully recovered, with improvements in their troponin levels, normal ECG, the improvement of echocardiography indices, and improvements in MRI indices at follow-up after a couple of months. Only a single 16-year-old patient, who remained without antibiotics, died 2 days after hospital admission [25].

In accordance with previous case reports, our patient was a young man; he demonstrated elevated levels of cardiac enzymes without there being any significant findings in the ECG and echocardiography. We would expect tachycardia in such a patient, with myocarditis, fever, and diarrhea. However, sinus bradycardia could be attributed to a combination of the direct inflammatory effects of myocarditis on the sinoatrial node, autonomic imbalance, and the possible influence of concurrent medication. Specifically, myocarditis could have directly affected the conduction system of the heart, including the sinoatrial node, leading to arrhythmias such as sinus bradycardia. This might have occurred due to localized myocardial inflammation or fibrosis in areas involved in impulse generation and conduction [32]. Moreover, the infection and subsequent systemic inflammation might have caused an imbalance in the autonomic nervous system, with excessive vagal tone predominating over sympathetic activity [33]. This is particularly plausible in *Campylobacter jejuni*-related infections, as molecular mimicry and autoimmune mechanisms can sometimes trigger atypical autonomic responses. Another significant reason may be that our patient was physically fit and had a naturally low resting heart rate, and thus, the fever and systemic inflammation might not have been sufficient to override their baseline sinus bradycardia [34]. Finally, our patient had a history of anxiety disorder and was receiving fluvoxamine. Selective serotonin reuptake inhibitors (SSRIs), including fluvoxamine, are known to occasionally cause bradycardia through vagal stimulation or other mechanisms [35,36]. This is a finding also highlighting the complexity of myocarditis presentations and the importance of comprehensive diagnostic evaluation in such cases.

Cardiac MRI did reveal myocardial edema and LGE, consistent with myocardial inflammation. Indeed, cardiac MRI plays a significant role in the diagnosis of myocarditis due to its ability to noninvasively assess myocardial structure, function, and tissue characterization [37]. Unlike traditional diagnostic methods, such as echocardiography or ECG, cardiac MRI provides detailed the visualization of myocardial inflammation, edema, and fibrosis using specific imaging techniques like T2-weighted imaging for detecting edema and LGE for identifying areas of necrosis or fibrosis [38,39]. These features are highly specific to myocarditis and allow differentiation from other conditions, such as acute coronary syndromes, without the need for invasive procedures like endomyocardial biopsy. The Lake Louise Criteria, established for cardiac MRI, provide standardized guidelines for diagnosing myocarditis by integrating findings from myocardial edema, hyperemia, and LGE [39]. This approach has significantly improved diagnostic accuracy, especially in patients with subtle or ambiguous clinical presentations. In the current case, cardiac MRI was instrumental in confirming the presence of myocardial inflammation and excluding ischemic injury, validating its role as a critical tool in the diagnostic workflow of myocarditis. Its noninvasive nature, combined with its ability to monitor disease progression and recovery, underscores the importance of cardiac MRI in modern cardiology.

EMB is often indicated in the diagnosis of myocarditis. However, it was not performed in this case because it was not deemed necessary based on current clinical guidelines and the patient’s clinical presentation. The clinical presentation of our patient was mild, with no hemodynamic instability, arrhythmias, or severe ventricular dysfunction, making EMB unnecessary, according to the American Heart Association (AHA) and European Society of Cardiology (ESC) guidelines. Moreover, EMB has a low sensitivity for detecting bacterial pathogens like *Campylobacter jejuni* in infectious myocarditis due to its limited capability in myocardial sampling. Another reason is that EMB is an invasive procedure with risks, including bleeding, cardiac perforation, arrhythmias, and infection. Given the patient’s stable clinical course and the clear diagnostic evidence from noninvasive tests, the risks of EMB outweighed the potential benefits. Finally, our patient responded well to supportive care and targeted antibiotic therapy, confirming the adequacy of the treatment plan without EMB [37,40,41,42].

Our patient was treated according to the latest guidelines, receiving a three-day course of 500 mg of azithromycin q.d. The treatment of *Campylobacter jejuni*-associated myocarditis with azithromycin is primarily based on the antibacterial activity of macrolides against *Campylobacter jejuni* and their ability to address systemic infection. Azithromycin is considered a first-line therapy due to its high efficacy against *Campylobacter jejuni*, its favorable safety profile, and its pharmacokinetics, which allow for high tissue penetration and prolonged intracellular activity [43]. This is particularly important in systemic infections, as azithromycin effectively targets the pathogen while reducing inflammation. In cases of myocarditis, azithromycin not only eradicates gastrointestinal infection but also mitigates systemic inflammation that may contribute to myocardial damage. The decision to treat with azithromycin is supported by clinical guidelines recommending antibiotics in cases of invasive *Campylobacter* infections or in patients with severe or extraintestinal manifestations, such as myocarditis [37,40,41,42]. In the current case, a three-day course of 500 mg daily resulted in the clinical resolution of symptoms and the normalization of cardiac biomarkers, highlighting its efficacy. The empirical use of azithromycin is especially relevant when antimicrobial susceptibility testing is unavailable, especially when other antibiotics, such as fluoroquinolones, present high resistance in *Campylobacter jejuni* [44].

The patient showed full recovery and was discharged after 8 days of hospitalization. His follow-up depicted no residual myocardial dysfunction at 1 month, and another appointment has been scheduled at 3 and 6 months post-illness. Furthermore, the patient was advised to avoid high-intensity physical activity for at least six months post-diagnosis, as exertion during this period could exacerbate myocardial stress and increase the risk of complications such as sudden cardiac death. He should return to normal physical activity only whether imaging and functional studies confirm complete recovery. Follow-up care for *Campylobacter jejuni* myocarditis is essential in monitoring recovery and detecting any potential complications such as residual myocardial dysfunction or arrhythmias. Typically, patients are advised to undergo serial evaluations, including clinical assessments, cardiac biomarker testing, and imaging. Transthoracic echocardiography is recommended within 1 to 3 months after discharge to confirm the normalization of cardiac function. Additionally, repeat cardiac MRI, usually performed at 3 to 6 months, can help ensure the resolution of myocardial edema and inflammation, as indicated by reduced late gadolinium enhancement [37,40,41,42].

Several differential diagnoses derived from symptoms such as acute chest pain, diarrhea, and elevated cardiac troponin, were considered. Chest pain with elevated troponins was highly suggestive of acute coronary syndrome in this patient. However, the patient’s young age was not convincing, his ECG showed no significant ischemic changes, cardiac MRI did not reveal features of ischemic myocardial injury (no subendocardial or transmural late gadolinium enhancement typical of infarction), and the patient had no significant cardiovascular risk factors, nor a family history of premature coronary disease. Pericarditis, which could overlap with myocarditis, was excluded as there was no pericardial effusion on echocardiography or cardiac MRI, no characteristic ECG findings such as widespread ST-elevation or PR depression were observed, and the chest pain was non-positional and lacked a pericardial friction rub. Acute chest pain with nonspecific ECG changes and elevated cardiac biomarkers could indicate pulmonary embolism, but it was also excluded due to normal oxygen saturation in room air, a lack of clinical symptoms such as significant dyspnea or tachypnea, and the absence of suggestive findings on echocardiography. Septic shock causing cardiac dysfunction was rejected because the patient was hemodynamically stable throughout hospitalization, with no signs of shock or multi-organ dysfunction. Finally, other types of myocarditis including viral, autoimmune, and toxic myocarditis were excluded, as the stool culture confirmed *Campylobacter jejuni* as the causative pathogen, while nasopharyngeal SARS-CoV-2 and syndromic multiplex respiratory PCR tests were negative.

The exact pathophysiology mechanism behind the myocardial involvement of *Campylobacter jejuni* still remains unknown. A possible hypothesis involves direct bacterial invasion, where *Campylobacter jejuni* reaches the myocardium through systemic dissemination from the gastrointestinal tract [11]. However, a more plausible mechanism is immune-mediated myocarditis triggered by molecular mimicry [7]. *Campylobacter jejuni* possesses antigens that share structural similarity with host cardiac proteins, potentially leading to cross-reactive immune responses and subsequent myocardial inflammation. In addition, toxins produced by *Campylobacter jejuni*, such as cytolethal distending toxin, may contribute to systemic inflammation and myocardial injury [11]. These toxins can disrupt cellular processes, leading to oxidative stress, cytokine release, and myocardial damage [11]. The involvement of these pathways is supported by the myocardial edema and inflammation observed in imaging studies [38]. The multifactorial nature of *C. jejuni*-induced myocarditis underscores its rarity and complexity, with the immune system playing a significant role in its pathogenesis.

The novelty of our manuscript lies in the integration of an atypical case presentation into a comprehensive systematic review, offering a unique opportunity to observe trends and variations in this infection. Specifically, this systematic review identifies that *Campylobacter jejuni*-related myocarditis predominantly affects young male individuals. This synthesis not only identifies commonalities across cases but also highlights diagnostic challenges in cases with minimal findings on standard testing such as ECG and echocardiography. Furthermore, our manuscript offers a robust treatment and follow-up framework, emphasizing the utility of cardiac MRI and evidence-based management protocols, which are often underreported in the literature.

### Limitations of the Study

This study provides valuable insights into *Campylobacter jejuni*-related myocarditis. However, a significant limitation is the absence of direct evidence demonstrating the presence of *Campylobacter jejuni* in the myocardium of the patient who was diagnosed with myocarditis. This omission raises the possibility of co-infecting agents, which can complicate the interpretation of findings. While EMB with PCR analysis is the gold standard for identifying viral genomes or other infectious agents, its invasive nature may limit its use. The absence of EMB in this case constrains the ability to definitively attribute myocardial inflammation solely to *Campylobacter jejuni*. Moreover, while noninvasive diagnostic methods, including cardiac MRI, strongly suggest myocardial inflammation, they cannot exclude the possibility of additional pathogens. Future studies should address this gap through direct myocardial sampling or advanced imaging with pathogen-specific biomarkers to confirm causality.

## 4. Conclusions

In conclusion, myocarditis related to *Campylobacter jejuni* appears to be a plausible diagnosis in patients who present to the emergency department with diarrhea and chest pain. Clinicians should maintain a high degree of suspicion when evaluating differential diagnoses, particularly in younger patients who exhibit elevated cardiac enzyme levels, chest pain, and symptoms of gastroenteritis. In such cases, heightened awareness and a targeted evaluation to rule out myocarditis are essential, alongside appropriate diagnostic tests for stool pathogens. Patients who receive timely antibiotic therapy tailored to the causative agent and supportive care generally recover fully within the first week of hospitalization. The diagnostic process in this case underscores the pivotal role of advanced imaging techniques, particularly cardiac magnetic resonance imaging, in confirming myocarditis in patients with subtle clinical presentations and non-specific findings on electrocardiograms or echocardiography. By outlining a structured approach to diagnosis, treatment, and follow-up, this report emphasizes the importance of evidence-based management strategies and highlights the necessity of considering uncommon causes in young patients presenting with chest pain and diarrhea. This comprehensive framework contributes valuable insights to the understanding and clinical management of this rare condition.

## Figures and Tables

**Table 1:** Demographics, troponin levels, imaging findings, antibiotics, and outcomes in all case reports of *Campylobacter jejuni*-related myocarditis found in the literature.

Case Report	Number of Patients	Age (Years)	Sex (M/F)	Peak Cardiac Troponin	ECG	Echocardiography	CMR	Antibiotics	Outcomes
Suehiro W et al., 2023 [12]	1	20	Male	12,794.7 pg/mL (n.r. 1–25 pg/mL)	Slightly elevated ST segments in leads I, II, aVF, V5, and V6	Decreased systolic left-ventricular function with diffuse hypokinesis and an LVEF of 40%, no pericardial effusion	LGE in the inferior wall and high-intensity area on T2-weighted image in part of the inferior wall	No	- LVEF improved from 40% to 57% - Myocardial edema in inferior wall of left ventricle was a little improved from 8 to 6 mm
Mohamed Jiffry MZ et al., 2023 [13]	2	35	Male	160 ng/L (n.r. <22 ng/L)	Nonspecific ST segment elevation in leads V2-V5	LVEF 60–65% with no regional wall motion abnormalities and no pericardial effusion	No	Azithromycin 500 mg q.d. Metronidazole 500 mg b.i.d.	- Resolution of the ST segment elevations - ESR and CRP normalized
		21	Male	818 ng/L (n.r. <22 ng/L)	ST segment elevation in leads II, III, and aVF with reciprocal ST depressions in leads I and aVL	Borderline-normal systolic function, LVEF 50–55%, focal areas of severe hypercontractility in the inferior, inferolateral, and lateral walls, severely hypocontractile apex and apical segments, no pericardial effusion	No	No	- Discharged the next day
Kojima N et al., 2022 [14]	1	13	Male	NA	Normal	LVEF = 75% without other dilatations in any chambers	Diffuse LGE at the epicardium	No	- Full recovery
Belfeki N et al., 2022 [15]	1	23	Male	678 ng/L (n.r. <14 ng/L)	Left-axis deviation with regular sinusal tachycardia	LVEF 55% with normal wall motions, no valvular dysfunction, normal pulmonary pressure and no pericardial effusion	Focal areas of hypersignal in the subepicardium of the posterolateral LV wall indicating myocardial edema, early hypersignal in the subepicardium of the posterolateral LV wall indicating focal hyperemia, subepicardial nodular lesions of myocardial damage	Azithromycin 1 g	- CRP and cardiac enzymes normalized after three weeks. - Repeated TTE and 24 h ECG were normal - Control CMR imaging at 3 months showed regression of the focal areas of hyper signal in the subepicardium of the posterolateral LV wall
Chantzaras AP et al., 2021 [16]	1	13	Female	456.4 pg/mL (n.r. <14 pg/mL)	Normal	LVEF >65% and no pericardial effusion	No markers of myocardial inflammation or necrosis	Azithromycin (initial dose of 10 mg/kg followed by 5 mg/kg once)	- Full recovery
Yaita S et al., 2020 [17]	1	16	Male	1.71 ng/mL	ST elevation in leads II, aVF, V3, V4, V5, and V6	Normal	No	No	- Discharged on Day 6 with no abnormalities in the ECG on Day 10 and troponin levels at 0.007 ng/mL on Day 17
Daboussi O et al., 2020 [9]	1	25	Male	136 ng/mL (n.r. 0–14 ng/mL)	Normal	Normal, LVEF 57%	Reduced LV systolic function (45%), areas of increased signal intensity on T2-weighted images suggesting myocardial edema, delayed enhancement of the lateral wall	Erythromicin 2 g daily	- Full recovery - Troponin I and CPK decreased to 93 U/L and 87 ng/mL, respectively
Greenfield GM et al., 2018 [18]	1	31	Male	402 ng/L	Normal	Normal	Normal LV volumes and function with localized myocardial edema and contrast enhancement within the basal inferolateral wall	No	- Full recovery
Obafemi MT et al., 2017 [19]	1	25	Male	1963 ng/L (n.r. <14 ng/L)	ST depression in anteroseptal lead	Mildly impaired LV systolic function, hypokinetic basal to mid-inferior septum and posterolateral wall, no pericardial effusion	No	- Meropenem 1 g 8 hourly IV - Switched to oral Ciprofloxacin 750 mg 12 hourly after 48 h	- Improvement in left-ventricular systolic function after 1 week - Full recovery
Gutiérrez de la Varga L et al., 2017 [20]	1	55	Male	676 (maximum 35)	NA	Normal	Presence of edema and enhancement without delay	Clarithromycin	- Discharge after a week
Panikkath R et al., 2014 [21]	1	43	Male	1.75 ng/mL (n.r. up to 0.03 ng/mL)	Mild ST segment elevations in the lateral leads without T wave inversions	LVEF 68%, with wall motion abnormalities in the inferior septum	Subepicardial and midmyocardial enhancement in the anterolateral wall and interventricular septum	Azithromycin 500 mg p.o. daily	- Discharge after 3 days - Full recovery
De Cock D et al., 2012 [11]	3	42	Male	15.6 μg/L (n.r. <0.13 μg/L)	Repolarization disturbances in the inferolateral leads	Moderately decreased systolic LV function with diffuse hypokinesia	Reduced LV systolic function (LVEF 40%), myocardial edema, diffuse and persisting enhancement of the subepicardium and the midwall	Azithromycin	- Partial recovery - Pathological late wall enhancement on cMR still visible but less pronounced
		34	Male	8.9 μg/L (n.r. <0.13 μg/L)	Elevated ST segments in leads V4-V6	Diffuse hypokinesia and moderately decreased LV function (EF 40%)	LGE confined to the subepicardium of the inferolateral wall, small pericardial effusion, global systolic LV function mildly decreased (EF 50%) with hypokinesia in the affected segments	No	- Normal exercise capacity - LV systolic function has recovered to normal (LVEF 62%), but focal areas of pathological late enhancement remain visible
		21	Male	11.6 μg/L (n.r. <0.13 μg/L)	Elevated ST segments in leads V4-V6	Moderately decreased systolic LV function (LVEF 40%)	Mild enlargement of both ventricles, small pericardial effusion, myocardial edema in the lateral wall of the LV, LGE of the subepicardium	Ciprofloxacin	- Full recovery after 1 month - Normal CMR except for late wall enhancement, which is still visible
Kratzer C et al., 2010 [22]	1	19	Male	0.52 ng/mL	Sinus tachycardia, strain on the right side of the heart with right-axis deviation, S wave in lead I and Q wave in lead III, and signs of myocardial injury with ST segment elevations in posterior and lateral leads	Severe hypokinetic area at the apex region	Spotted hyperenhancement on the late enhancement sequences in the area of the left ventricle from the heart base to the medial third; inferoseptal unctum maximum in lateral, inferolateral, and circumscribed directions	Ciprofloxacin IV	- Discharge on day 15 - The ECG showed a normofrequent sinus rhythm, left-axis deviation, a slow R-progression in V1–V3, and T-inversion in I, aVL, and V5–V6 - Normal global systolic function without any wall motion abnormalities - Complete resolution of the CMR signs of inflammation after 3 weeks
Turley AJ et al., 2008 [23]	1	24	Male	1.4 ng/mL (n.r. <0.01 ng/mL)	ST elevation in leads V1-V4, II	Small pericardial effusion (<1 cm) with an akinetic LV apical segment and an abnormal appearance of the LV apex	Patchy gadolinium enhancement	Erythromycin	- Full recovery - Improvement in LV systolic function - Normal exercise tolerance - Discharge after 14 days
Mera V et al., 2007 [24]	1	43	Male	Positive (levels NA)	ST segment elevation, along with isodifasic T wave, in leads I, aVL, and V4-V6	Uncertain apical akinesis	No	Clarithromycin 500 mg b.i.d.	- Full recovery
Pena LE et al., 2007 [25]	1	16	Male	NA	NA	NA	No	No	- Death after 2 days
Hannu T et al., 2005 [26]	1	43	Male	NA	Depression of inferolateral ST segments	Normal	No	Erythromycin	- Discharge the next day with normal ECG - Full recovery after 2 months
Hamdulay SS et al., 2004 [27]	1	34	Male	0.59 ng/L	Sinus tachycardia with ST segment elevation in leads V2–V4	Significant LV impairment (LVEF 30%) with antero-apical hypokinesia	No	Erythromycin	- Dyspnoea improved, pyrexia settled, improvement in liver function tests, inflammatory markers, and renal biochemistry, which had all normalized by the 10th day of admission - Normal ECG and LVEF after 3 months
Cunningham C et al., 2003 [28]	1	30	Male	30.2 µg/L (n.r. 0–0.5 µg/L)	T wave inversion in the lateral and inferior leads	Normal	No	Ciprofloxacin 500 mg bid	- Full recovery - Discharge after 5 days
Wanby P et al., 2001 [29]	1	26	Male	58 μg/L (n.r. up to 1.0 μg/L)	Inferolateral ST wave elevations	Normal	No	No	- Discharge after 6 days with fever, diarrhea, and chest pains having disappeared and ECG wave changes having decreased - At follow-up, a normal ECG, echocardiogram, and ECG exercise test
Coc ID et al., 2001 [30]	1	32	Male	NA [elevated CPK and CK-MB]	Low atrial rhythm with a mean frontal QRS axis of 30 degrees and symmetrically inverted T waves in leads V4-6, SI, and aVL	Dilated LV [LVIDd 6.8 cm, LVIDs 6.0 cm] with globally impaired systolic function and mild mitral regurgitation	Diffuse enhancement in myocardial signal intensity following the IV administration of gadolinium–DTPA; active inflammation in the septum and lateral wall	Gentamycin	- Gradual improvement in his exercise tolerance - Reduction in LV dimensions [LVIDd 6.5 cm, LVIDs 4.4 cm] at 6-month follow-up - Significant reduction in the enhancement of myocardial signal intensity suggesting resolving myocardial inflammation in CMR after 3 months
Florkowski CM et al., 1984 [31]	1	23	Male	NA [elevated CPK and CK-MB]	ST changes and T wave inversion in the chest leads	Normal	No	No	- Discharge after 3 days - Normal ECG after 9 weeks - Normal symptom-limited maximal treadmill stress test after 9 weeks

LGE, late gadolinium enhancement; q.d., quaque die; b.i.d., bis in die; IV, intravenous; LVEF, left-ventricular ejection fraction; LVIDd, left-ventricular internal diameter in diastole; LVIDs, left-ventricular internal diameter in systole; ESR, erythrocyte sedimentation rate; CRP, C-reactive protein; CPK, creatine phosphokinase; CK-MB, creatine kinase-MB; TTE, transthoracic echocardiogram; ECG, electrocardiogram; aVF, augmented vector foot; aVL, augmented vector left; ST, segment between the S wave and T wave; CMR, cardiac magnetic resonance; n.r., normal range; NA, not available.

## Data Availability

Data are available upon request.
